# Identification of Beef Odors under Different Storage Day and Processing Temperature Conditions Using an Odor Sensing System

**DOI:** 10.3390/s24175590

**Published:** 2024-08-29

**Authors:** Yuanchang Liu, Nan Peng, Jinlong Kang, Takeshi Onodera, Rui Yatabe

**Affiliations:** 1 Research and Development Center for Five-Sense Devices, Kyushu University, 744 Motooka, Nishi-ku, Fukuoka 819-0395, Japan; 2 Graduate School of Information Science and Electrical Engineering, Kyushu University, 744 Motooka, Nishi-ku, Fukuoka 819-0395, Japan

**Keywords:** beef quality, food safety, odor sensing system, artificial olfactory system, sensor array, non-destructive testing, UMAP

## Abstract

This study used an odor sensing system with a 16-channel electrochemical sensor array to measure beef odors, aiming to distinguish odors under different storage days and processing temperatures for quality monitoring. Six storage days ranged from purchase (D0) to eight days (D8), with three temperature conditions: no heat (RT), boiling (100 °C), and frying (180 °C). Gas chromatography–mass spectrometry (GC-MS) analysis showed that odorants in the beef varied under different conditions. Compounds like acetoin and 1-hexanol changed significantly with the storage days, while pyrazines and furans were more detectable at higher temperatures. The odor sensing system data were visualized using principal component analysis (PCA) and uniform manifold approximation and projection (UMAP). PCA and unsupervised UMAP clustered beef odors by storage days but struggled with the processing temperatures. Supervised UMAP accurately clustered different temperatures and dates. Machine learning analysis using six classifiers, including support vector machine, achieved 57% accuracy for PCA-reduced data, while unsupervised UMAP reached 49.1% accuracy. Supervised UMAP significantly enhanced the classification accuracy, achieving over 99.5% with the dimensionality reduced to three or above. Results suggest that the odor sensing system can sufficiently enhance non-destructive beef quality and safety monitoring. This research advances electronic nose applications and explores data downscaling techniques, providing valuable insights for future studies.

## 1. Introduction

As an essential meat product, beef is an integral part of global culinary traditions due to its high nutritional value, diverse cooking methods, and significant economic and cultural impact [1,2,3]. Consequently, beef food safety has garnered widespread attention, and the demand for higher beef quality is increasing [4,5]. The preservation state of retail beef is usually assessed through its microbial contents, supplemented by physicochemical indicators such as color and pH [6]. Sometimes, sensory analysis is conducted, where trained panels evaluate the beef’s odor, taste, and tenderness [7,8]. The microbial content is a crucial indicator of the beef preservation status and potential contamination. With more extended processing and storage days, the likelihood of increased microbial contents in refrigerated beef grows [9].

Microbial content analysis requires the direct sampling of beef and can provide corroborating evidence of spoilage and contamination [10,11]. However, the testing time is lengthy and can hardly be used for all beef products. The shelf life after packaging can often be developed based on experimental data [12,13]. Beef is considered safe to consume when stored at the specified temperature and with intact packaging. Safety precedes taste in terms of shelf life, so consumers have less chance of obtaining more information about the beef quality. Moreover, some near-expiry beef may be processed into cooked products to extend its sale time. Although heating can inhibit microbial growth, extended storage reduces beef’s flavor and nutritional value, complicating consumer choices for quality beef.

Therefore, determining the beef quality under specific conditions, particularly assessing safety, is an important yet unresolved issue. Rapid, non-destructive methods for assessing beef quality or its preservation status are needed. Non-destructive methods include assessing meat odor substances and evaluating the beef flavor through optical techniques such as near-infrared spectroscopy [14,15,16,17,18]. Beef odor is a significant indicator of its freshness and spoilage state. Fresh beef has a mild odor, while spoiled beef emits a distinct foul or sour smell due to microbial contamination [19,20].

Many research groups have developed electronic noses or artificial olfactory systems based on various principles to quickly detect odor characteristics [21,22,23,24,25,26,27,28]. Due to the diverse range of odor substances and the ongoing clarification of their receptive characteristics, these systems often use sensor arrays and machine learning techniques for analysis. Consequently, the application of dimensionality reduction methods and classification algorithms within machine learning has become a crucial aspect of data analysis in sensor array research [29,30]. The trends of electronic noses in recent years are covered in several excellent reviews (e.g., [31,32]). Electronic noses based on various principles show good selectivity in distinguishing different food categories [33,34]. However, electronic noses are often affected by humidity [35,36,37]. Detecting high-moisture foods is challenging and requires design considerations across sensor arrays, measurement systems, and data analyses.

In our previous study, an electronic nose based on an electrochemical receptor sensor array, also referred to as an odor sensing system, was developed [37,38]. The odor sensing system uses a 16-channel sensor array with grid-shaped electrodes coated with gas-absorbing materials. These materials are composed of gas chromatography stationary-phase materials (GC materials) mixed with carbon black (CB) (a fine black powder composed of carbon). The GC materials absorb odor molecules and undergo volumetric changes, while the carbon black imparts conductivity, causing the overall resistance of the sensitive material to change with the volume [39,40]. The odor sensing system, with its non-destructiveness and rapid detection, has recently been used for detection in various fields, such as the deterioration of transformer insulation oil in production safety and the odors of overheated electrical wires in home safety, providing early fire warnings [41,42].

In this study, we used an odor sensing system to address the issue of beef food safety detection by distinguishing odors. This investigation was intended to identify beef odors under different storage conditions through machine learning. We focused on two variables: storage days and temperature. For the processing temperatures, we set three conditions: no heat, boiling (100 °C), and frying (180 °C), to identify the beef quality after heating and processing. The future vision of this study is to develop a non-destructive and non-contact method for beef quality management and detection based on odor.

We also used gas chromatography–mass spectrometry (GC-MS) to analyze the odor components of beef and to understand the extent of the odor changes under different conditions. Given the high moisture content of beef samples, we developed an experimental system with a humidity control module to verify whether the odor sensing system can effectively capture odor characteristics under humidity influence. We used six commonly used machine learning classifiers in the data analysis and compared two different dimensionality reduction methods: principal component analysis (PCA) and uniform manifold approximation and projection (UMAP).

## 2. Materials and Methods

### 2.1. Beef Samples

The beef samples used in this study were ground beef of Japanese origin, purchased from a popular supermarket. The consumption period was set to three days after the purchase date. We designated the purchase date as storage day zero (D0), with additional storage durations set as D0, D1, D2, D4, D6, and D8. After purchase, we mixed all the beef together and divided it into 5 g portions, each placed in a 20 mL capped glass sample vial. The samples were stored at 4 °C until the designated days.

The processing temperatures were set to three conditions: no heating (room temperature, RT), 100 °C, and 180 °C. The no-heating condition represented the odor of raw meat, 100 °C simulated boiling conditions, and 180 °C simulated frying conditions. After reaching the designated storage days, the refrigerated beef samples were subjected to temperature operations for 10 min to ensure that the center of the samples reached the set temperature. The samples were heated in capped vials placed in a heater equipped with aluminum blocks. After heating, the samples were cooled to room temperature before measurement. Compositional analysis and odor measurement used the same preparation method.

In addition, the beef samples caused the humidity sensor (sensirion SHT35) next to the sensor array of the odor sensing system to reach approximately 70% humidity during the measurement process. To eliminate the influence of humidity, we also tested reference gases with adjusted humidity at 70% (humid air) as a comparison. Therefore, there are 19 categories of samples.

### 2.2. GC-MS Analysis

The beef odor components were analyzed using a Shimadzu GC-MS QP2010-SE, with a WAX 30 m × 0.25 mm × 0.25 μm column (Agilent Technologies, Santa Clara, CA, USA). Helium was used as the carrier gas. The injector temperature was set to 240 °C, with a split-injection mode (1:5 ratio) and a constant flow rate of 40 cm/s. The oven temperature program was from 40 °C (3 min) to 230 °C (8 min) at a heating rate of 10 °C/min. The mass spectrometer used a quadrupole analyzer with an interface temperature of 240 °C and an ion source temperature of 200 °C. Sampling was performed using solid-phase microextraction (SPME) with Agilent’s SPME Arrow PDMS 100 μm fiber. We used an automatic sampling system (PAL system, Agilent) to absorb the sample at room temperature for 600 s before injection into the GC-MS.

### 2.3. Odor Sensing System

The computer unit, analog signal converter board, and 16-channel sensor chip of the odor sensing system were provided by Panasonic Industry Corp (Tokyo, Japan). The GC materials of the sensor array are shown in Table 1. In the table, the McReynolds constant represents the polarity of the GC materials [43,44]. CB was sourced from Sigma-Aldrich Japan G.K. The GC materials and CB were mixed using tetrahydrofuran (THF) (Sigma-Aldrich, St Louis, MO, USA) as the solvent applied to the 16-channel sensor array chip using a reagent spotting system (SPOT MASTER, Musashi Engineering, Tokyo, Japan). After one hour of annealing at 170 °C, the sensor array was installed onto the system’s baseboard in a polytetrafluoroethylene (PTFE) chamber.

### 2.4. Experimental System

**Figure 1:**
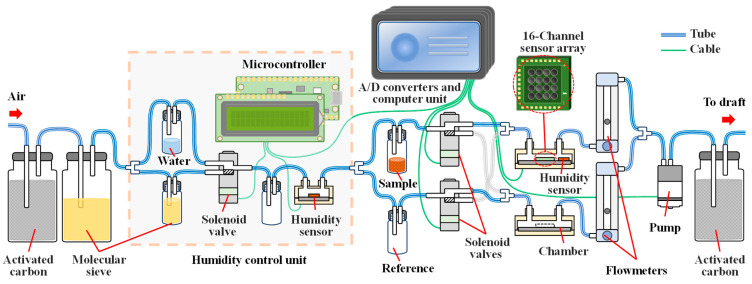
Experimental system of the odor sensing system.

The measurement system used in this study is illustrated in Figure 1. Room temperature during the experiments was controlled at 25 °C by the air conditioner. Laboratory air was used as the carrier gas, filtered through a 4A molecular sieve and an activated carbon filter. Humidity was adjusted using a humidity control module controlled by a microcontroller. Humidity was controlled by adjusting the ratio of air passing through molecular sieves and water-filled bottles using solenoid valves, maintaining a relative humidity of 30%. The flow of the sample and reference gases was switched using solenoid valves. The flow rate was set to 0.15 L/min, controlled by a gas pump and flow meter. Each channel maintained continuous flow during measurements, with symmetrical flow paths allowing smooth gas switching while maintaining flow rates. The computer module recorded the sensor array signals through an analog-to-digital converter (ADC). Exhaust gases were filtered through activated carbon and vented to a fume hood.

### 2.5. Measurement Conditions

We collected odor data from the beef samples using the odor sensing system. Each condition had five beef samples, with each sample subjected to three measurement cycles. Therefore, there were a total of 15 measurements for the beef odor samples under each condition. Each measurement cycle consisted of 40 s of sample gas followed by 80 s of reference gas. The reference gas flow was set to twice the sample gas flow duration to allow the sensor chip adequate time to regain equilibrium. The data sampling rate was set to 100 times per second, resulting in each measurement having 12,000 data points per 16 channels, totaling 192,000 dimensions. Additionally, each measurement began and ended with an extra 20 s of reference gas to confirm the sensor status and address boundary issues during data smoothing.

### 2.6. Data Analysis

#### 2.6.1. Preprocessing Techniques

Initial data smoothing was performed using the moving average method to eliminate noise. The Fast Fourier Transform was computed for each data cycle, and the 15 frequencies with the highest intensities were selected as features. A total of 240 features were extracted from the 16-channel sensor array data. The machine learning code was written using Python 3.10.12.

#### 2.6.2. Dimensionality Reduction

Feature reduction was further achieved using PCA and UMAP. PCA is a linear dimensionality reduction method that is particularly effective for data with linear relationships. UMAP is a manifold learning method designed to dimensionalize high-dimensional data by finding low-dimensional manifold structures [45]. The UMAP can find and preserve the nonlinear structure of the data. We reduced the number of features from 240 dimensions to a range of 2–12 dimensions. Both supervised and unsupervised UMAP conditions were compared to evaluate their effectiveness at clustering data.

#### 2.6.3. Machine Learning

Classifiers used to identify beef odor samples under different conditions included a support vector machine (SVM) with a linear kernel, a K-nearest neighbors (KNN) classifier, a decision tree (DT) classifier, a random forest (RF) classifier, a multilayer perceptron (MLP) classifier, and a logistic regression (LR) classifier. The classifiers were implemented using the scikit-learn library, version 1.2.2.

## 3. Results and Discussion

### 3.1. Detection of Beef Odorant Substance Composition Using GC-MS

The GC-MS experimental results presented in Table 2 reveal a novel insight into the components of beef odor. A diverse range of components, such as alkanes, alcohols, aldehydes, ketones, acids, and esters, were detected. The odor types of the components shown in Table 2 were cross-referenced from the Good Scents company webpage (www.thegoodscentscompany.com) and the National Library of Medicine webpage (www.ncbi.nlm.nih.gov).

Some significant odor compounds were found only in beef samples with a storage day of D0. These compounds in Table 2, including compounds No. 9 (2-Nonen-1-ol, green), No. 17 (pentanal, pungent), No. 27 (2,3-Pentanedione, buttery), and No. 35 (2,3-Octanedione, buttery), provide valuable insights into the unique odor profile of fresh beef.

In beef samples with a storage day of D4, the variety of odor compounds was simpler. Compound No. 31 (acetoin), which had a buttery smell, showed the highest detection intensity. Additionally, No. 7 (1-Pentanol, pungent) and No. 8 (1-Hexanol, sweet) also had relatively high intensities.

By day D8, the number and variety of odor compounds had increased significantly. The detection intensities of No. 31 (acetoin) and No. 8 (1-Hexanol) became stronger. Similarly, the No. 7 (1-Pentanol) levels increased, and No. 41 (ammonium acetate), which has an ammonia-like odor, was detected.

From the heating temperature perspective, beef odors under RT conditions exhibited fewer types of components. Compounds such as No. 30 (2-Heptanone), No. 15 (Butanal, 2-methyl-), and No. 33 (2-Propanone, 1-Hydroxy-), which have fruity odors, were more easily detected at 100 °C or 180 °C.

Components No. 7 (1-Pentanol) and No. 18 (hexanal), which has a grassy odor, showed relatively high detection intensities at 100 °C. Pyrazines typically have a nutty odor, and furans, with sweet or woody odors, were detected at 180 °C. Additionally, some furans were detected under other temperature conditions on day D8.

Furthermore, specific odor components with larger detection intensities, such as No. 18 (hexanal), No. 7 (1-Pentanol), No. 31 (acetoin), and No. 8 (1-Hexanol), which may play a dominant role, showed a more significant correlation with the storage days than with the temperature changes. Specifically, hexanal and No. 7 (1-Pentanol) exhibited a tendency to decrease and then increase with the storage duration, while acetoin and No. 8 (1-Hexanol) tended to increase gradually over days.

In summary, the GC-MS analysis results indicate that the changes in the beef odor compounds are relatively significant. Under varying storage day and processing temperature conditions, these odor changes provide objective criteria for differentiation.

### 3.2. Identification of Beef Odor Using Artificial Olfactory System

#### 3.2.1. Response of Sensor Arrays to Beef Samples

**Figure 2:**
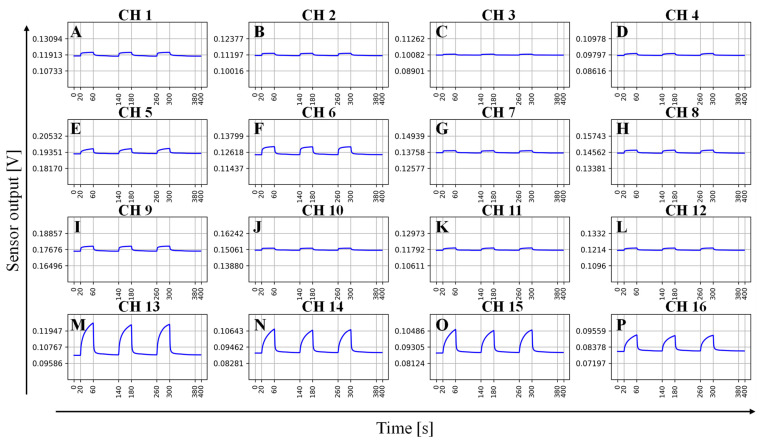
Odor sensing system 16-channel sensor array responses of a beef sample. (**A**) Apiezon L; (**B**) SE-30; (**C**) OV-1; (**D**) DC-11; (**E**) SE-52; (**F**) OV-3; (**G**) DC-550; (**H**) DC-710; (**I**) OV-17; (**J**) Tween 80; (**K**) OV-210; (**L**) Siponate DS-10; (**M**) PEG 1000; (**N**) PEG 600; (**O**) OV-275; (**P**) PEG 2000.

Figure 2 shows a beef sample undergoing three cycles of measurement. After exposing the sensor array to the sample for 40 s, it was flushed with reference gas for 80 s, which was sufficient to restore equilibrium.

#### 3.2.2. Visualization Using Dimensionality Reduction Methods

- PCA Visualization

**Figure 3:**
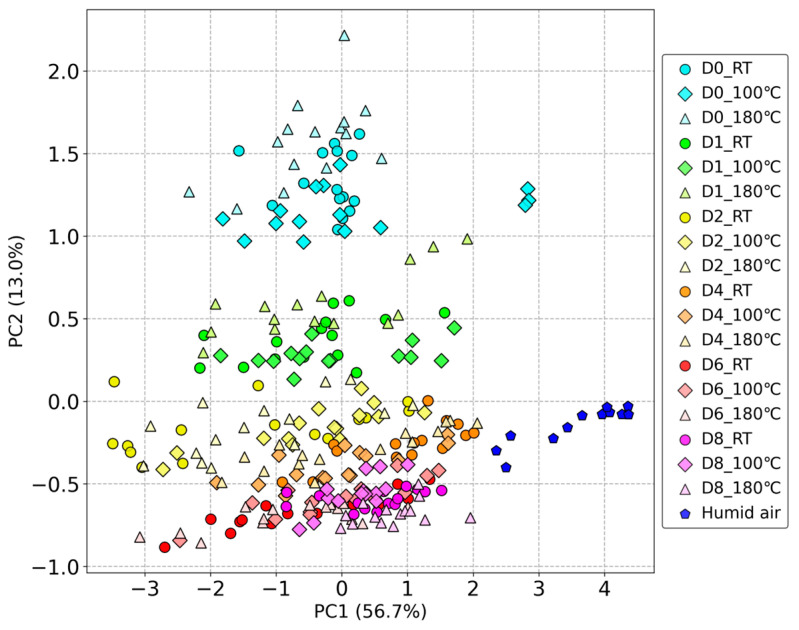
PCA dimensionality reduction results of beef odor measured by odor sensing system.

After feature extraction, the 240-dimensional feature data were visualized using PCA dimensionality reduction, as shown in Figure 3. We normalized the data using Min–Max Normalization before the dimensionality reduction. The results of the PCA show that on the right side of PC1 are the reference gases adjusted for humidity, while on the left are the beef odor samples. In PC2, the distribution of the data from bottom to top reflects the changes in storage days. There is a clear separation between D0 and D1 and other dates, while on D6 and D8, the data points are intermixed within the same region. The differences due to different processing temperatures can be observed to some extent but are not as significant as the differences in storage days. The cumulative contribution rate of PC1 and PC2 is 69.7%, indicating a considerable loss of information when reducing the data to two dimensions using PCA. Higher-dimensional data may be needed for better classification results.

- Unsupervised UMAP

After optimizing the parameters of the UMAP using a grid search, the following parameters were used: number of neighbors: 140; minimum distance: 0.8; metric: Euclidean distance; and random seed: 42.

**Figure 4:**
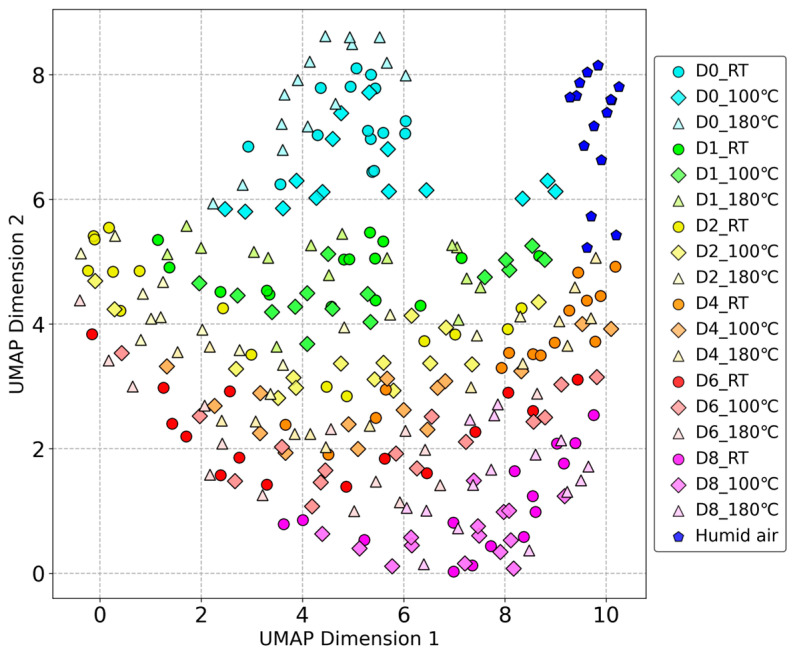
Unsupervised UMAP dimensionality reduction results of beef odor measured by odor sensing system.

The results of the dimensionality reduction using unsupervised UMAP are shown in Figure 4. There is a correspondence between the data distribution in the PCA and that in the unsupervised UMAP. The results of the reference gases adjusted for humidity also maintained a certain distance from the beef odor samples. Compared to PCA, UMAP provides better differentiation between storage days, and the data are distributed according to the storage duration. The differences, particularly between D4 and D8, are more pronounced. However, the clustering of different processing temperatures did not improve compared to PCA.

- Supervised UMAP

**Figure 5:**
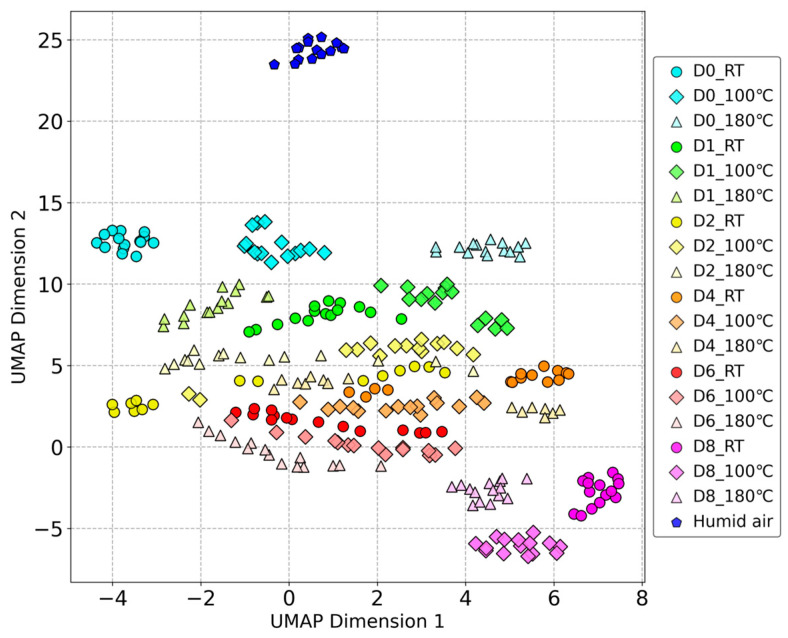
Supervised UMAP dimensionality reduction results of beef odor measured by odor sensing system.

Figure 5 shows the results of the supervised UMAP dimensionality reduction. The parameters for the supervised UMAP were the same as those for the unsupervised UMAP. The input consisted of the answers of 19 different sample categories, and the target weight was set to 0.4 to avoid overfitting (the default value was 0.5).

Supervised methods generally achieve better clustering effects than unsupervised methods because they incorporate category information during dimensionality reduction. Figure 5 shows a greater distance between the humid air and beef odor samples. The different storage days and processing temperatures can be easily distinguished in the results. Each category has its own centralized distribution areas, which is beneficial for classification. Nonetheless, some mixed points can be observed in the results, for conditions are partially clustered from D2 to D4.

#### 3.2.3. Beef Odor Recognition by Classifiers

The main parameters of each classifier were optimized using a grid search. The parameter settings for each classifier are shown in Table 3. Additionally, the number of iterations for the SVM, LR, and MLP was set to 5000. The random seed for all the classifiers was set to 42. Other parameters not explicitly mentioned were set to their default values.

The results of the classification using the classifiers after PCA and UMAP dimensionality reduction are shown in Table 4. Accuracy rates were calculated by leave-one-out cross-validation (LOOCV). The dimensions (Dim) after dimensionality reduction were set to 2, 3, 4, 5, 6, 9, and 12. The cumulative explained variances (CEVs) for the PCA are also listed in Table 4. The CEV is also known as the cumulative contribution ratio. The CEV represents the proportion of the original data’s variance explained by the PCA results after dimensionality reduction.

The recognition accuracies of the PCA and unsupervised UMAP are approximately 40–50%, both at the same level. The recognition accuracy of the supervised UMAP is over 90%. Each method shows a trend of increasing recognition accuracy as the number of dimensions increases.

When using PCA for dimensionality reduction, SVM and LR perform better than other classifiers. PCA achieves an accuracy of 57.2% at six dimensions, and the CEV is 82.9%. As a comparison, SVM achieves an accuracy of 45.3% at two dimensions with a 69.7% CEV. The accuracy was improved by about 12% with the increasing dimensions and higher CEV values. Further increasing the number of dimensions does not yield better recognition results. It can be argued that using PCA methods to reduce the dimensionality to six dimensions is more effective.

After unsupervised UMAP dimensionality reduction, SVM achieves the highest recognition accuracy of 49.1% at four dimensions. Higher-dimensional reductions do not result in higher accuracy. This can also be corroborated by the visualization results of the PCA and UMAP, where the differences in storage days are more significant than the differences in processing temperatures. In the classification results after PCA and unsupervised UMAP dimensionality reduction, misjudgments are more likely to occur between different processing temperatures on the same storage date.

For supervised UMAP dimensionality reduction, KNN, RF, and MLP achieve accuracies of over 99.0% at two dimensions. At three dimensions, the recognition accuracy can reach over 99.5%, representing a balance between the computational cost and recognition precision. The recognition accuracies of SVM, DT, and LR reach 92.3%, 93.0%, and 83.9% for the two dimensions, respectively, and improve with the increasing dimensionality. Different classifiers exhibit a certain degree of variability. All classifiers stabilize at 9 or 12 dimensions with recognition accuracies above 95.0%. After dimensionality reduction using supervised UMAP, the classifiers based on different principles yielded consistent classification results. All six classifiers effectively classified the beef samples, reflecting the reliability of the dataset under different classifiers. Therefore, the supervised UMAP significantly enhances the classification accuracy. Therefore, the odor sensing system and analytical methods used in this study can effectively identify beef odors on different storage days and at different processing temperatures.

## 4. Conclusions

In this study, we used a 16-channel electrochemical sensor array to measure beef odors, aiming to distinguish odors under different storage day and processing temperature conditions for quality monitoring. Six storage days ranged from the purchase date (D0) to eight days (D8), with three temperature conditions: no heat (RT), boiling (100 °C), and frying (180 °C).

GC-MS analysis revealed that while the types of odor substances in the beef remained similar across the different storage states, their concentrations varied. Compounds like acetoin and 1-Hexanol significantly changed with the storage days, indicating their potential as markers for beef freshness. Pyrazines and furans were more detectable at higher temperatures (100 °C and 180 °C), highlighting the differences in beef odors under various storage conditions.

Data from the odor sensing system were visualized using PCA and UMAP. PCA and unsupervised UMAP distinguished beef odor samples from humidity-adjusted air, especially for different storage days, but struggled with the processing temperatures. Supervised UMAP accurately clustered different temperatures and dates.

The SVM and LR classifiers achieved 57% accuracy with PCA-reduced data. Unsupervised UMAP combined with SVM yielded 49.1% accuracy, with further parameter adjustments showing no improvement. Misjudgment is more likely between different processing temperatures on the same storage date. In contrast, supervised UMAP significantly enhanced the classification accuracy, achieving over 99.5% with the dimensionality reduced to three or above, enabling the system to manage both date and temperature variations. The results suggest that the odor sensing system has the potential to enhance the food safety and quality management of beef in a non-destructive way.

The supervised nonlinear dimensionality reduction method UMAP played an important role in the classification results. However, in order to confirm whether the nonlinear method is superior to the linear method, more method comparisons are needed in the future. Furthermore, the study was limited to one type of beef, and we suggest including more varieties in future research to validate the results. We also aim to optimize the sensor array by examining material selectivity in future studies. In particular, we aim to enhance the performance of recognizing food products with high humidity with different categories and not only beef. Enhancing the sensor array’s response characteristics based on odor components and exploring different receptor materials and processing methods could improve data collection and the iterative design of the odor sensing system, advancing our understanding of odor recognition principles.

## Figures and Tables

**Table 1:** GC materials of the 16-channel sensor array.

Channel	Material	McReynolds Constant	Resistance (KΩ)
CH 1	Apiezon L	143	1.32
CH 2	SE-30	217	1.23
CH 3	OV-1	222	1.09
CH 4	DC-11	276	1.05
CH 5	SE-52	334	2.32
CH 6	OV-3	423	1.40
CH 7	DC-550	620	1.56
CH 8	DC-710	827	1.66
CH 9	OV-17	884	2.08
CH 10	Tween 80	993	1.72
CH 11	OV-210	1520	1.29
CH 12	Siponate DS-10	1720	1.34
CH 13	PEG 1000	2587	1.11
CH 14	PEG 600	2646	0.98
CH 15	OV-275	4219	0.96
CH 16	PEG 2000	2353–2587	0.86

**Table 2:** Odor components of beef detected by GC-MS.

No.	Time (min)	Component	Odor Type	Peak Intensity (×10^3^)								
				Day 0			Day 4			Day 8		
				RT	100 °C	180 °C	RT	100 °C	180 °C	RT	100 °C	180 °C
Alkanes												
1	1.55	Heptane	Sweet, ethereal	-	-	20.2	-	-	-	-	56.7	20.0
2	1.84	Octane	Odorless	-	-	45.1	-	-	-	81.8	75.3	35.7
Alcohols												
3	3.04	Ethanol	Fresh, slightly sweet	13.5	11.6	-	-	-	-	61.9	39.4	20.2
4	6.55	1-Penten-3-ol	Herbal, floral	-	-	-	-	-	-	20.0	29.4	
5	6.55	1-Butanol	Alcohol-like	-	-	-	-	-	-	25.4	26.8	
6	7.61	1-Butanol, 3-methyl-	Strong, fusel oil-like	-	-	-	-	-	-	110.8	19.8	13.6
7	8.30	1-Pentanol	Pungent, fusel oil-like	49.1	37.2	29.6	17.9	21.0	12.5	187.1	221.8	79.4
8	9.87	1-Hexanol	Fresh, sweet, fruity	12.2	-	14.3	25.5	24.1	15.3	280.0	384.0	114.3
9	10.53	2-Nonen-1-ol	Green, cucumber-like	-	-	24.5	-	-	-	-	-	-
10	11.23	1-Octen-3-ol	Mushroom-like	19.9	15.7	18.8	-	-	-	86.2	101.2	52.2
11	11.30	1-Heptanol	Musty, herbal, sweet	-	-	-	-	-	-	21.3	20.2	-
12	12.64	1-Octanol	Fatty, citrus	-	-	-	-	-	-	13.1	11.7	-
13	13.38	2-Octen-1-ol, (Z)-	Fresh, green	-	-	-	-	-	-	-	13.9	-
14	13.82	1-Nonen-4-ol	Green, cucumber-like	-	-	-	-	-	-	16.6	27.9	-
Aldehydes												
15	2.74	Butanal, 2-methyl-	Fruity	-	-	43.4	-	-	15.2	11.2	-	167.0
16	2.88	Butanal, 3-methyl-	Fruity	-	-	132.1	-	-	51.0	-	23.0	473.6
17	3.73	Pentanal	Pungent, penetrating	-	14.7	-	-	-	-	-	-	-
18	6.20	Hexanal	Fresh, green, grassy	127.9	178.5	87.6	-	31.9	28.8	-	365.5	242.1
19	7.29	Heptanal	Fruity, oily	-	11.1	24.2	-	-	-	-	30.3	51.5
20	8.99	Octanal	Fatty, citrus-like	-	-	30.2	-	-	-	-	-	-
21	10.50	Nonanal	Waxy, citrus	-	-	-	-	-	-	-	14.2	17.2
22	11.53	Furfural	Sweet, almond-like	-	-	-	-	-	-	-	-	30.1
23	12.36	Benzaldehyde	Bitter almond	-	-	-	-	-	-	-	-	48.3
Ketones												
24	1.95	Acetone	Sweet, fruity	-	-	-	-	-	-	51.1	59.4	-
25	2.65	2-Butanone	Sweet, acetone-like	-	-	14.2	-	-	-	-	-	60.1
26	5.11	Acetylpropionyl	Butter-like	-	-	-	-	-	-	-	-	25.9
27	5.13	2,3-Pentanedione	Buttery	13.9	-	14.5	-	-	-	-	-	-
28	3.72	2,3-Butanedione	Strong, buttery	-	-	-	-	-	-	-	22.0	33.6
29	6.91	Heptan-2-one	Fruity, banana-like	-	-	-	-	-	-	15.7	29.7	-
30	7.21	2-Heptanone	Fruity, banana-like	-	-	30.1	-	-	11.5	-	-	131.0
31	8.92	Acetoin	Butter-like	15.4	11.5	-	61.8	80.2	48.4	202.4	736.6	699.2
32	9.02	2-Propanone, 1-methoxy-	Mild, sweet	-	-	19.0	-	-	-	-	-	-
33	9.47	2-Propanone, 1-hydroxy-	Mild, sweet	-	-	30.7	-	-	-	-	-	136.4
34	9.50	2,5-Octanedione	Sweet, buttery	-	-	-	-	-	-	-	26.7	43.9
35	9.52	2,3-Octanedione	Sweet, buttery	44.8	-	-	-	-	-	-	-	-
36	11.53	2-Propanone, 1-(acetyloxy)-	Mild, sweet	-	-	12.5	-	-	-	-	-	27.5
Acids												
37	13.56	Butanoic acid	Unpleasant, rancid, buttery	-	-	-	-	-	-	13.7	-	-
38	16.04	Hexanoic acid	Fatty, cheesy	-	-	-	-	-	-	41.2	15.7	-
Esters												
39	1.95	Allyl acetate	Fruity	-	-	-	-	-	11.7	-	-	-
40	2.00	1-Propen-2-ol, acetate	Fruity, floral	-	-	27.3	-	-	-	-	-	108.9
41	11.30	Ammonium acetate	Ammonia-like	-	-	-	-	-	-	216.0	-	-
42	21.25	Diethyl phthalate	Odorless	47.1	-	-	-	-	-	165.3	-	15.2
Hydrocarbons												
43	2.08	1-Octene	Mild, waxy	-	-	-	-	-	-	-	11.9	-
44	8.25	Styrene	Sweet, balsamic	-	-	-	-	-	-	14.0	-	-
Pyrazines												
45	7.74	Pyrazine	Nutty, roasted	-	-	17.3	-	-	-	-	-	64.1
46	8.56	Pyrazine, methyl-	Nutty, roasted	-	-	-	-	-	-	-	-	175.6
47	9.55	Pyrazine, 2,5-dimethyl-	Nutty, roasted	-	-	26.1	-	-	-	-	-	50.5
48	9.61	Pyrazine, ethyl-	Nutty, roasted	-	-	-	-	-	-	-	-	80.1
49	9.78	Pyrazine, 2,3-dimethyl-	Nutty, roasted	-	-	-	-	-	-	-	-	19.4
50	10.35	Pyrazine, 2-ethyl-5-methyl-	Nutty, roasted	-	-	-	-	-	-	-	-	38.5
51	10.64	Pyrazine, trimethyl-	Nutty, roasted	-	-	15.5	-	-	-	-	-	30.3
Furans												
52	2.41	Furan, 2-methyl-	Sweet, woody	-	-	11.6	-	-	-	-	-	43.6
53	3.34	Furan, 2-ethyl-	Sweet, woody	-	-	29.0	-	-	18.1	-	31.7	198.3
54	6.33	Furan <2-butyl->	Sweet, woody	-	-	-	-	-	-	-	-	17.4
55	8.06	Furan, 2-pentyl-	Sweet, woody	-	-	65.5	-	-	27.3	26.6	45.7	430.1
56	13.69	Furan, tetrahydro-2-methyl-	Sweet, nutty	-	-	-	-	-	-	35.6	43.1	47.9
57	13.95	2-Furanmethanol	Sweet, almond-like	-	-	78.8	-	-	12.7	-	-	164.5
Others												
58	4.40	Thiophene	Unpleasant, garlic-like	-	-	-	-	-	-	-	-	13.3
59	5.58	Thiophene, 2-methyl-	Unpleasant, garlic-like	-	-	-	-	-	-	-	-	16.2
60	8.60	Pyrimidine, 2-methyl-	Unpleasant, garlic-like	-	-	49.0	-	-	19.9	-	-	-
61	12.24	Pyrrole	Sweet, caramel-like	-	-	14.2	-	-	-	-	-	82.0

Gradient color scales are used to represent peak intensity values. Lower values are represented by green, medium values by yellow, and higher values by red.

**Table 3:** The parameters for each classifier.

Abbreviation	Classifier	Parameter
SVM	Support Vector Machine	kernel = “linear”, C = 100
KNN	K-Nearest Neighbors	n_neighbors = 15, weights = “distance”, metric = “minkowski”
DT	Decision Tree	max_depth = 8, criterion = “log_loss”, splitter = “random”
RF	Random Forest	max_depth = 8, criterion = “log_loss”, n_estimators = 100
MLP	Multilayer Perceptron	hidden_layer_sizes = 6, activation = “tanh”, solver = “lbfgs”
LR	Logistic Regression	C = 100

**Table 4:** Accuracy rates of beef odor recognition using different classifiers.

	Dim	Accuracy of Classifiers [%]						CEV [%]
		SVM	KNN	DT	RF	MLP	LR	
PCA	2	45.3	44.6	40.0	44.9	43.5	42.1	69.7
	3	49.1	47.0	44.9	48.1	46.0	49.1	73.7
	4	56.1	44.2	42.5	48.8	52.6	52.6	77.2
	5	55.1	44.9	36.1	48.4	50.2	53.7	80.3
	6	57.2	46.0	39.7	46.0	50.5	57.2	82.9
	9	55.1	48.4	40.4	48.8	41.1	55.8	88.7
	12	55.1	44.9	40.0	46.0	40.4	53.7	92.4
Unsupervised UMAP	2	39.7	41.1	37.5	37.9	40.0	36.1	-
	3	43.9	40.4	24.9	35.8	40.7	43.2	-
	4	49.1	42.8	28.4	42.1	44.2	44.6	-
	5	44.6	37.5	34.7	39.0	38.3	42.1	-
	6	43.5	39.3	30.5	40.7	37.5	41.1	-
	9	45.6	39.0	33.3	39.0	40.4	44.2	-
	12	41.4	40.7	32.3	44.9	37.5	41.8	-
Supervised UMAP	2	92.3	99.0	93.0	99.3	99.3	83.9	-
	3	99.7	98.3	94.0	100.0	99.7	97.5	-
	4	99.0	98.6	93.0	98.6	96.5	97.9	-
	5	100.0	99.7	90.9	99.7	99.3	100.0	-
	6	100.0	98.6	91.6	100.0	98.6	99.3	-
	9	100.0	99.7	97.9	99.7	99.0	100.0	-
	12	100.0	99.0	96.8	99.7	96.1	100.0	-

## Data Availability

The data presented in this study are available upon request.
